# Pushing the limit of stability

**DOI:** 10.1093/nsr/nwae265

**Published:** 2024-07-30

**Authors:** Donglong Fu, Guodong Sun, Jinlong Gong

**Affiliations:** School of Chemical Engineering & Technology, Key Laboratory for Green Chemical Technology of Ministry of Education, Tianjin University; Collaborative Innovation Center for Chemical Science & Engineering (Tianjin), China; International Joint Laboratory of Low-carbon Chemical Engineering, China; School of Chemical Engineering & Technology, Key Laboratory for Green Chemical Technology of Ministry of Education, Tianjin University; Collaborative Innovation Center for Chemical Science & Engineering (Tianjin), China; International Joint Laboratory of Low-carbon Chemical Engineering, China; Joint School of National University of Singapore and Tianjin University, International Campus of Tianjin University, China; School of Chemical Engineering & Technology, Key Laboratory for Green Chemical Technology of Ministry of Education, Tianjin University; Collaborative Innovation Center for Chemical Science & Engineering (Tianjin), China; International Joint Laboratory of Low-carbon Chemical Engineering, China; Joint School of National University of Singapore and Tianjin University, International Campus of Tianjin University, China

Catalyst stability plays a crucial role in chemical processes due to its economic, environmental and safety implications. A stable catalyst reduces replacement costs, preserves product quality, and minimizes waste generation and energy consumption. Therefore, a key consideration in catalyst design is achieving superior stability while maintaining high catalytic efficiency and selectivity. This is particularly relevant in the on-purpose production of propylene through the propane dehydrogenation (PDH) process, where Pt- and Cr-based catalysts have been commercialized by Lummus Technology and UOP Honeywell, respectively. Both catalysts face rapid deactivation due to coking, sintering and structural deterioration, necessitating frequent and expensive regeneration [1]. Decades of research have resulted in remarkable progress with regard to the design of new metal [2] and oxide catalysts [3] as well as chemical processes [4], yet these catalysts have not demonstrated functionality for longer than 500 hours under industrially relevant conditions.

Recently, as reported in *Science*, Zeng *et al.* created a In/Rh@Silicalite-1 catalyst capable of continuously producing propylene with 99% selectivity at near-equilibrium conversion for 5500 hours under a pure propane stream (Fig. 1) [5]. This achievement was enabled by the confinement of RhIn_4_ within the micropores of Silicalite-1 (S-1), the siliceous form of MFI zeolites. As demonstrated by exhaustive characterizations and DFT calculations, the key to this success lies in the migration of In atoms to the micropore-confined Rh clusters, resulting in the formation of single Rh sites in the form of RhIn_4_ anchored by silanol nests in S-1 [6]. At a propane conversion of 45% at 600°C, the propylene productivity of this catalyst is one to two orders of magnitude higher than that of traditional Pt-based catalysts at similar propane conversion rates. The RhIn_4_@S-1 catalyst also demonstrates high efficiency and stability in the dehydrogenation of ethane and n-butane, indicating significant potential for the development of greener and more cost-effective low-alkane dehydrogenation processes.

**Figure 1.**
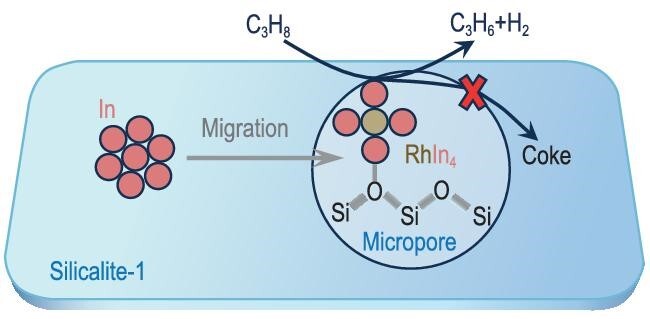
Schematic representation of the formation of the RhIn_4_@S-1 catalyst and its coking-resistant nature for PDH.

Benefiting from its coking-resistant nature, the RhIn_4_@S-1 catalyst enables hydrogen free PDH, which simplifies the overall process and reduces capital and operating costs. Besides, the absence of hydrogen not only enhances process safety but also boosts energy efficiency, as hydrogen production typically involves energy-intensive processes. Overall, this research has introduced a novel, energy-efficient catalytic system for dehydrogenation, reducing the need for regeneration—a notable departure from conventional Pt- and Cr-based catalysts for alkane dehydrogenation.

The research from Zeng *et al.* is expected to be of interest to both academia and industry. It remains to be seen whether this catalyst is stable for regeneration once the performance deteriorates as the single atom RhIn_4_ is stabilized by its deliberated interaction with silanol nests in S-1. The confinement nature of the catalyst will encourage studies on structural performance, including the effects of micropore size and shape [7,8]. Exploring new chemical processes, such as coupling this catalyst with selective hydrogen combustion materials, could further enhance its performance and energy efficiency [4]. The heat generated by the exothermic selective hydrogen combustion could potentially offset the energy required for the endothermic propane dehydrogenation process. If successful, this new catalyst has the potential to become a strong competitor to industrial Pt- and Cr-based catalysts.

## References

[1] Monai M, Gambino M, Wannakao S et al. Chem Soc Rev 2021; 50: 11503–29. 10.1039/D1CS00357G 34661210

[2] Chen S, Zhao Z, Mu R et al. Chem 2021; 7: 387–405. 10.1016/j.chempr.2020.10.008

[3] Chen S, Xu Y, Chang X et al. Science 2024; 385: 295–300. 10.1126/science.adp7379 39024431

[4] Wang W, Chen S, Pei C et al. Science 2023; 381: 886–90. 10.1126/science.adi3416 37498988

[5] Zeng L, Cheng K, Sun F et al. Science 2024; 383: 998–1004. 10.1126/science.adk5195 38422151

[6] Zhao D, Tian X, Doronkin DE et al. Nature 2021; 599: 234–8. 10.1038/s41586-021-03923-3 34759363 PMC8580824

[7] Liu L, Lopez-Haro M, Lopes CW et al. Nat Mater 2019; 18: 866–73. 10.1038/s41563-019-0412-6 31263227

[8] Fu D, van der Heijden O, Stanciakova K et al. Angew Chem Int Ed 2020; 59: 15502–6. 10.1002/anie.201916596 PMC7496746 32026555

